# A compact single-layer tri-band directional antenna for Wi-Fi 6/6E applications

**DOI:** 10.1371/journal.pone.0359290

**Published:** 2026-10-01

**Authors:** Anh Nguyen-Quang, Hung Tran-Huy, Hung Nguyen-Manh, Muhammad Ikram

**Affiliations:** 1 Faculty of Electrical and Electronic Engineering, PHENIKAA School of Engineering, PHENIKAA University, Hanoi, Vietnam; 2 Faculty of Electrical and Electronics Engineering, Hanoi Open University, Hanoi, Vietnam; 3 College of Engineering and Applied Sciences, American University of Kuwait (AUK), Safat, Kuwait; Galgotias College of Engineering and Technology, Greater Noida, INDIA

## Abstract

A compact single-layer, single-feed tri-band directional antenna is presented for Wi-Fi 6/6E systems. The target bands at 2.4, 5.2, and 6.0 GHz yield a highest-to-lowest frequency ratio of 2.50, making compact integration of the low-band radiator particularly challenging. To address this issue, the 2.4-GHz driven patch is reactively loaded with narrow capacitive gaps, shorting vias, and meandered sections, reducing its resonant length from 33 to 13 mm (approximately 61%). Two proximity-coupled parasitic patches generate the 5.2- and 6.0-GHz resonances. The spatially distinct current paths provide nearly independent control of the three operating bands. A prototype occupying 0.36λ × 0.36λ × 0.012λ, where λ is the free-space wavelength at 2.4 GHz, exhibits measured −10-dB impedance bandwidths of 20, 90, and 100 MHz and peak broadside realized gains of 3.1, 5.8, and 6.8 dBi, respectively. The proposed configuration provides a compact and structurally simple solution for channel-specific Wi-Fi 6/6E links.

## Introduction

Wireless local area networks (WLANs) have evolved from single-band links into multiband platforms serving dense and heterogeneous device populations. Wi-Fi 6, based on IEEE 802.11ax, improves spectral efficiency in the established 2.4- and 5-GHz bands, whereas Wi-Fi 6E extends the same air interface into the 6-GHz spectrum. The three bands provide complementary functions: 2.4 GHz offers extended coverage and legacy compatibility, 5 GHz supports higher-throughput links with reduced congestion, and 6 GHz provides additional spectrum for wider channels and increased network capacity. Consequently, a single antenna capable of supporting all three bands is attractive for compact access points, gateways, and fixed wireless terminals.

Printed monopoles and slot antennas are widely used for compact WLAN terminals because they are simple to fabricate and offer broad impedance bandwidth. Tri-band responses have been realized using slots, stubs, parasitic strips, and resonant loading, with reported operating bands spanning 2.4/3.5/5–8 GHz and gains from below 1.5 dBi to approximately 5.4 dBi [1–5]. More recent studies have extended operation toward Wi-Fi 6E and Wi-Fi 7 using folded monopoles, gap-coupled branches, slot-based MIMO arrays, flexible CPW-fed radiators, and printed inverted-F antennas [5–9]. However, these configurations generally produce omnidirectional or bidirectional patterns with moderate gain [10]. Such radiation is suitable for handheld terminals but is less effective for fixed links, for which broadside directivity can improve sector gain, front-to-back ratio, and link reliability.

Dielectric resonator antennas (DRAs) are also attractive candidates for directional radiation [11,12]; however, they typically exhibit a relatively high profile. Microstrip patch antennas are therefore attractive for low-profile directional WLAN devices because they provide planar integration and inherent broadside radiation [13–18]. Dual-band operation has been obtained using L-shaped slots, with gains of 4.99 and 6.25 dBi at 2.45 and 5.8 GHz [15] and using edge cuts together with matching and harmonic-suppression networks, with gains of 3.5 and 5.2 dBi at 2.45 and 5 GHz [14]. Higher gain can be achieved by introducing frequency-selective surfaces, metasurfaces, or additional reflecting structures, yielding approximately 6.5–7.5 dBi at 2.4/5.2–5.8 GHz [13,17,18]. These approaches provide effective directional radiation but commonly increase the footprint, profile, layer count, and fabrication complexity. Moreover, dual-band configurations do not simultaneously support the 2.4-, 5-, and 6-GHz Wi-Fi 6/6E bands.

Reported tri-band patch antennas frequently employ multilayer, stacked, or multiport configurations [19–24]. Single-layer alternatives reduce the profile [25–27], but their resonances can remain strongly coupled, so adjustment of one band may perturb the others. A further challenge arises when the highest-to-lowest frequency ratio is large: the resonators require substantially different electrical lengths yet must be accommodated within a common compact aperture. The resulting dimensional disparity complicates impedance matching, inter-resonator coupling, and preservation of consistent broadside radiation across the operating bands. Thus, a compact single-layer, single-feed tri-band patch antenna with nearly independent resonance control and a frequency ratio of approximately 2.5 remains difficult to realize.

This work proposes a compact tri-band directional antenna operating at 2.4, 5.2, and 6.0 GHz. The principal contributions are threefold: 1) the 2.4-GHz driven patch is miniaturized by combined capacitive and inductive loading, reducing its resonant length by approximately 61%; 2) two proximity-coupled parasitic patches create the 5.2- and 6.0-GHz resonances while enabling nearly independent tuning; and 3) the complete single-layer prototype occupies only 0.36λ × 0.36λ × 0.012λ, where λ is the free-space wavelength at 2.4 GHz, while maintaining broadside directional radiation in all three bands.

## Single-band radiator

### Antenna design

Fig 1 shows the top and cross-sectional views of the miniaturized low-band radiator. The radiator is printed on a Taconic RF-35 substrate with relative permittivity $\epsilon_{r}=3.5$ and thickness *h* = 1.52 mm, and the opposite surface is fully metallized as a ground plane. A conventional rectangular patch operating in the fundamental TM_10_ mode has a resonant length of approximately one-half of the effective wavelength. To reduce this length, narrow capacitive gaps and symmetric meandered sections are introduced into the patch, while four shorting vias connect selected locations of the radiator to the ground plane.

**Fig 1:**
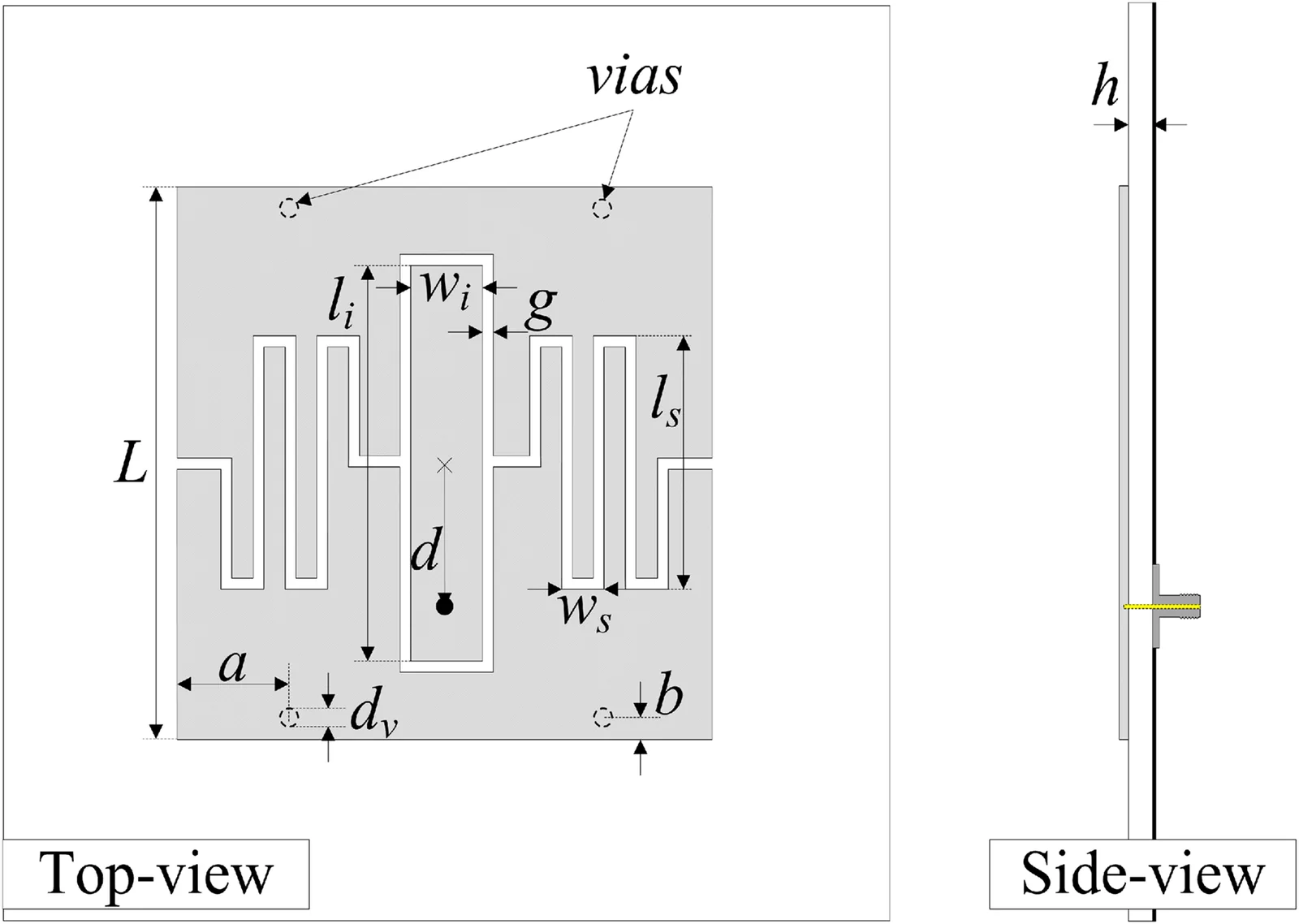
Geometry of the miniaturized low-band radiator.

The antenna was modeled and optimized using the High Frequency Structure Simulator (HFSS). The final dimensions are *h* = 1.52, *L* = 13, $l_{i}=9.4$, $w_{i}=1.8$, *g* = 0.22, *d* = 3.8, $l_{s}=6.0$, $w_{s}=1.0$, *a* = 2.7, *b* = 0.5, and $d_{v}=0.4$ mm.

### Antenna operation characteristics

To quantify the miniaturization, Fig 2 compares the simulated reflection coefficients of a conventional patch and the proposed loaded patch, both optimized for the same resonance. The conventional radiator requires a resonant length $L_{conv}=33$ mm, whereas the proposed design resonates with *L* = 13 mm, corresponding to a length reduction of approximately 61%. The stronger reactive loading inevitably increases the quality factor and therefore reduces the fractional impedance bandwidth and realized gain. Nevertheless, the measured 20-MHz lower-band bandwidth is adequate for a selected 20-MHz channel in the 2.4-GHz Wi-Fi 6 band.

**Fig 2:**
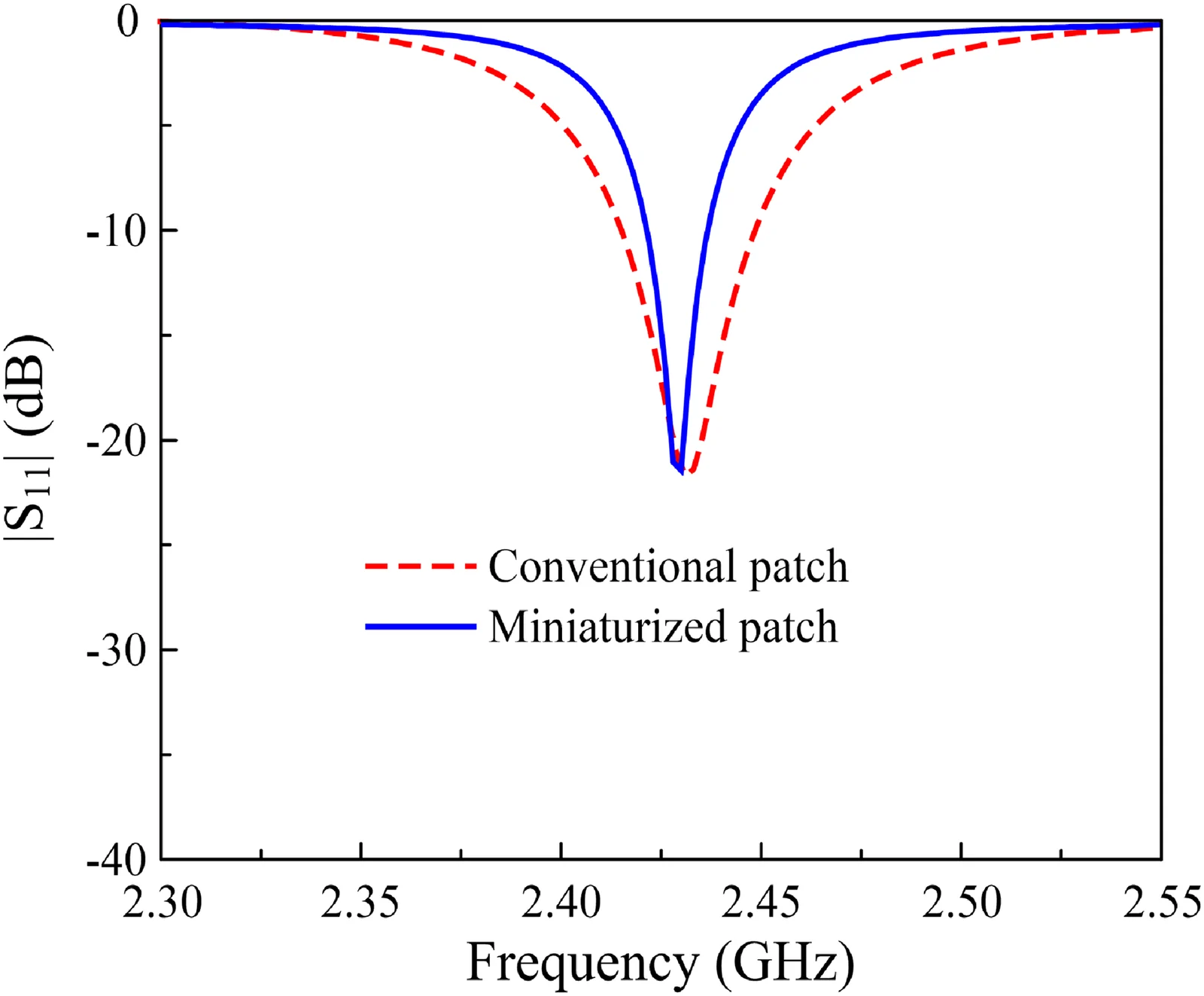
Simulated reflection coefficients of the conventional and miniaturized low-band radiators.

The resonance can be interpreted using the approximate lumped relation $f_{0}=1/(2\pi \sqrt{LC})$, where *L* and *C* denote the effective inductance and capacitance, respectively. Increasing either parameter reduces the resonant frequency. As discussed in [28], the narrow gaps supporting in-phase equivalent magnetic currents provide capacitive loading, whereas the meandered sections extend the current path and introduce additional inductive loading. Their combined effect therefore shifts the fundamental resonance downward without increasing the outer patch dimension.

The narrow slots embedded in the 2.4-GHz driven patch are not independent resonant slots. They form a reactive-loading network within a single radiator, rather than behaving as separate half-wavelength slot resonators. The gap width *g* primarily controls the capacitive loading, whereas the meander length $l_{s}$ governs the effective inductive loading. The parametric results in Fig 3 show that decreasing *g* increases the coupling capacitance and shifts the resonance to a lower frequency. Similarly, increasin*g* $l_{s}$ results in larger inductance and thus lowers the resonance. The field distributions in Fig 4 support this interpretation: strong electric fields are concentrated across the narrow gaps and along the meandered sections. The surface currents remain predominantly in phase over the radiator, confirming preservation of the fundamental broadside mode and vertically polarized radiation.

**Fig 3:**
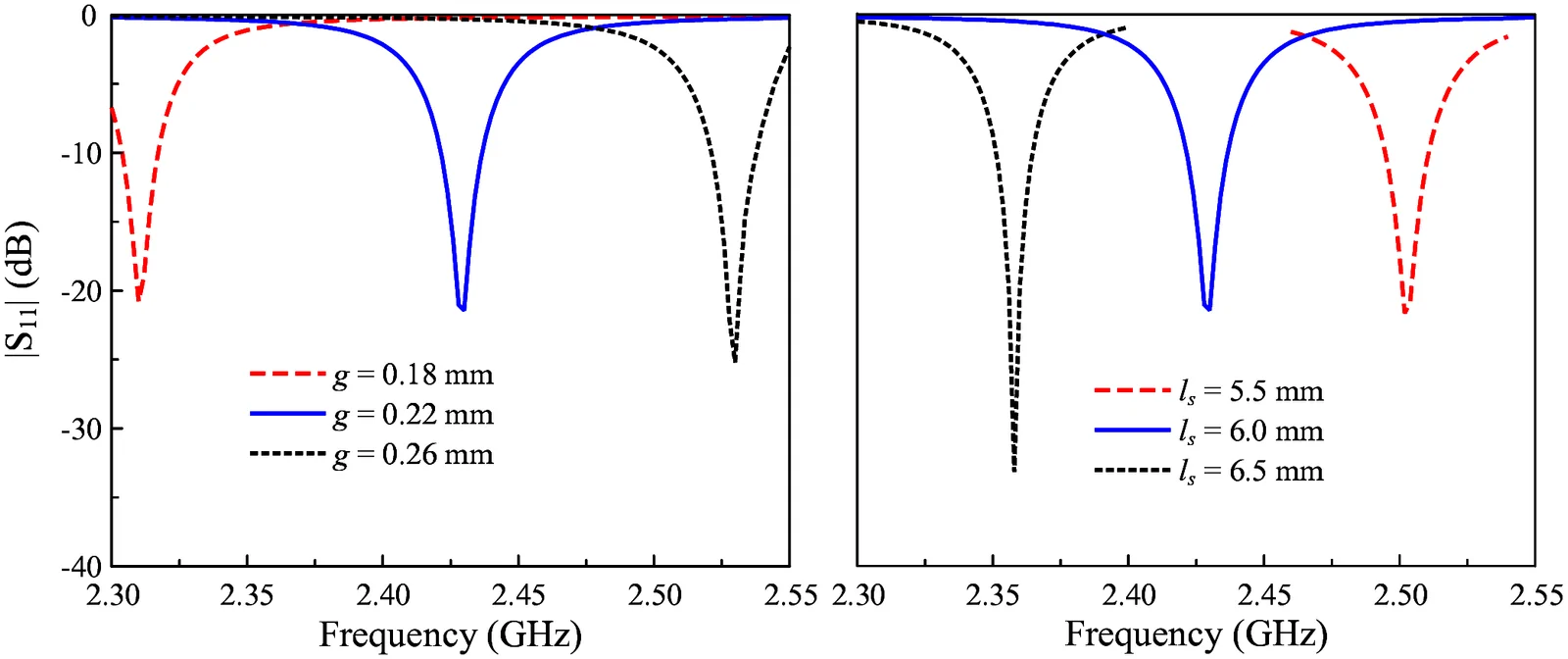
Effect of gap width *g* and meander length $l_{s}$ on the reflection coefficient of the miniaturized radiator.

**Fig 4:**
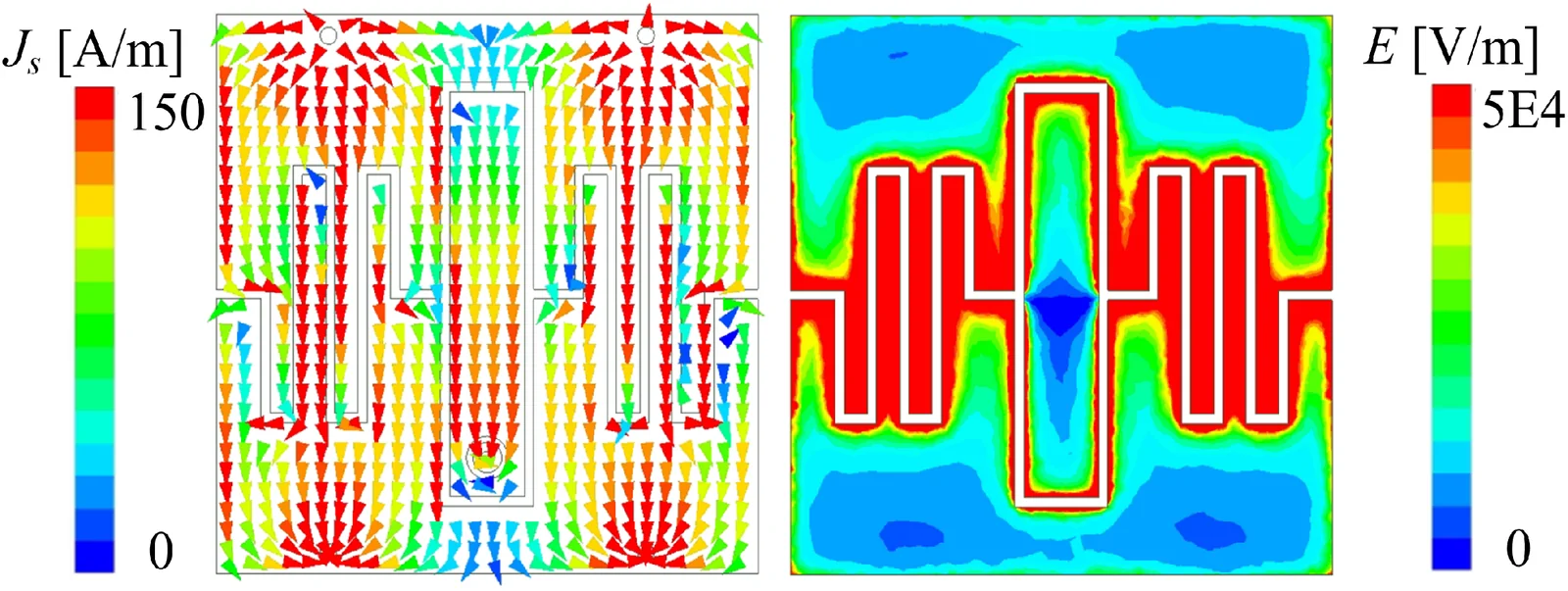
Simulated surface-current and electric-field distributions of the miniaturized low-band radiator.

## Development of the dual-band antenna

### Antenna configuration

The dual-band configuration in Fig 5 is developed from the miniaturized radiator of Section 2. The probe-fed central patch generates the 2.4-GHz resonance, while a rectangular parasitic patch is positioned adjacent to it to produce the 5.2-GHz mode through proximity coupling. The parasitic-patch length is initially selected near one-half of the effective wavelength at the target upper frequency. The optimized dimensions are *L* = 13, $l_{i}=9.4$, $w_{i}=1.8$, *g* = 0.22, *d* = 3.8, $l_{s}=6.0$, $w_{s}=1.0$, *a* = 2.7, *b* = 0.5, $d_{v}=0.4$, *s*_1_ = 0.3, *L*_1_ = 14.6, and *W*_1_ = 8 mm.

**Fig 5:**
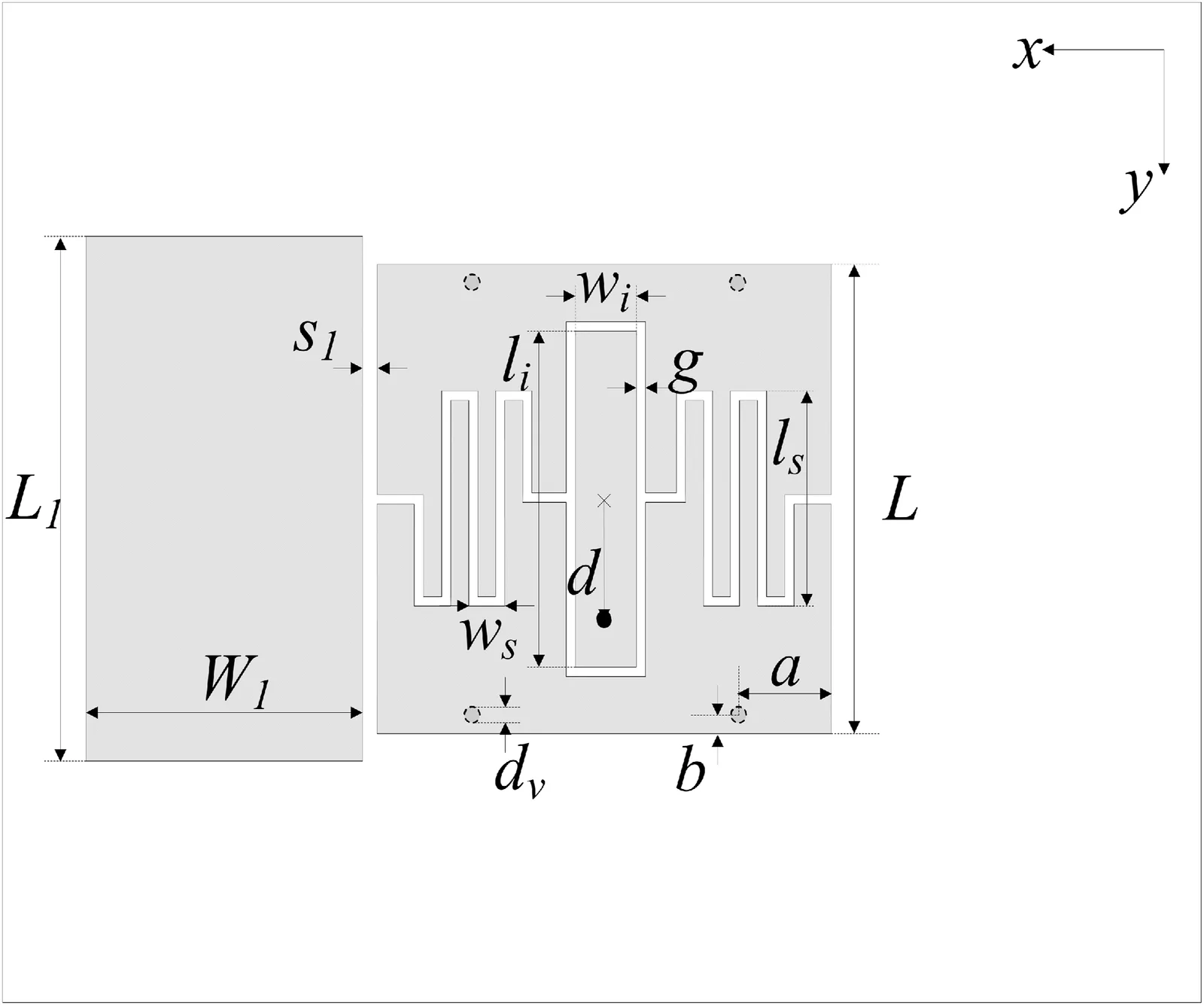
Geometry of the compact dual-band antenna.

### Simulated performance and independent tuning

Fig 6 presents the simulated reflection coefficient and peak realized gain of the dual-band antenna. The −10-dB impedance bandwidths are 2.41–2.43 GHz and 5.16–5.25 GHz, with corresponding peak realized gains of 3.3 and 5.8 dBi. The 3D radiation patterns in Fig 7 exhibit broadside directional beams at 2.42 and 5.22 GHz, demonstrating that the parasitic upper-band radiator does not compromise the desired radiation orientation.

**Fig 6:**
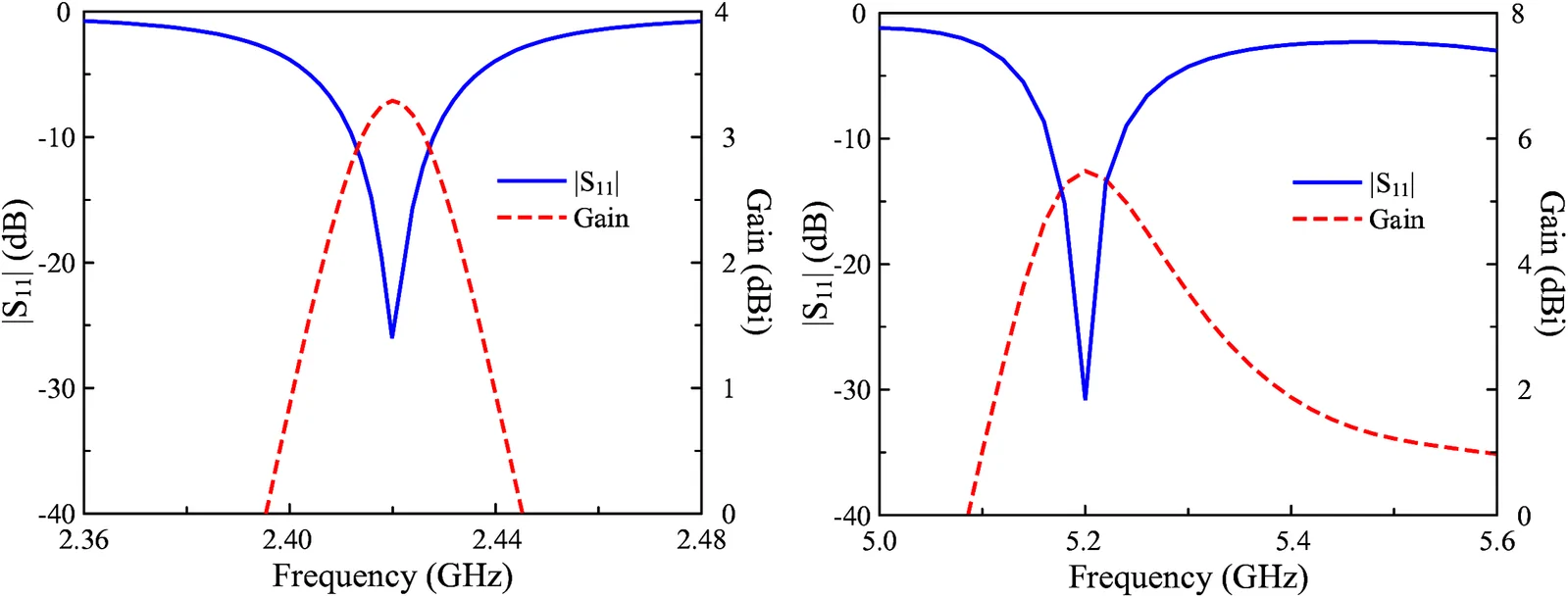
Simulated reflection coefficient and peak realized gain of the dual-band antenna for the low band (left) and high band (right).

**Fig 7:**
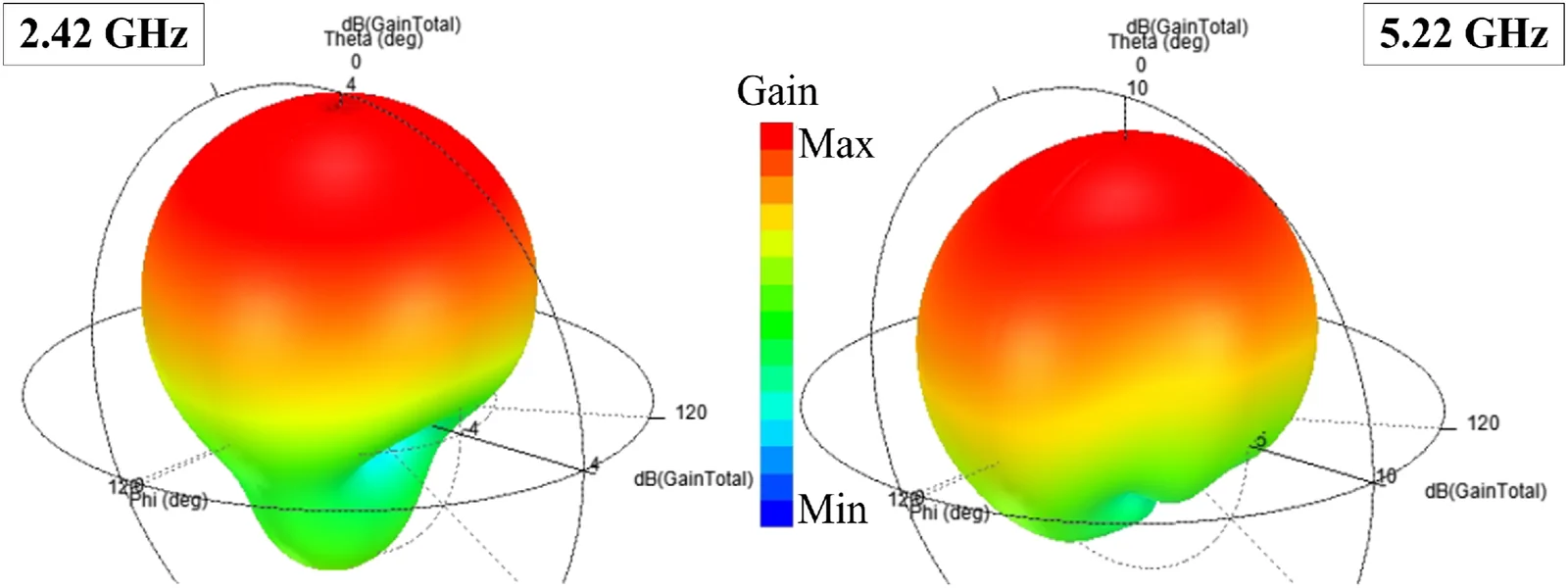
Simulated 3D radiation patterns of the dual-band antenna at 2.42 and 5.22 GHz.

Independent frequency control is evaluated through the parametric results in Figs 8 and 9. The lower resonance is determined mainly by the gap width *g* and meander length $l_{s}$ of the driven patch. By contrast, increasing the parasitic-patch length *L*_1_ predominantly shifts the upper resonance downward, while producing only a minor change in the 2.4-GHz response. This behavior confirms that the two resonances are associated with physically distinct current paths. As shown in Fig 9, the coupling gap *s*_1_ mainly controls the impedance matching around 5.2 GHz; its effect on the lower band is comparatively weak and is omitted for brevity.

**Fig 8:**
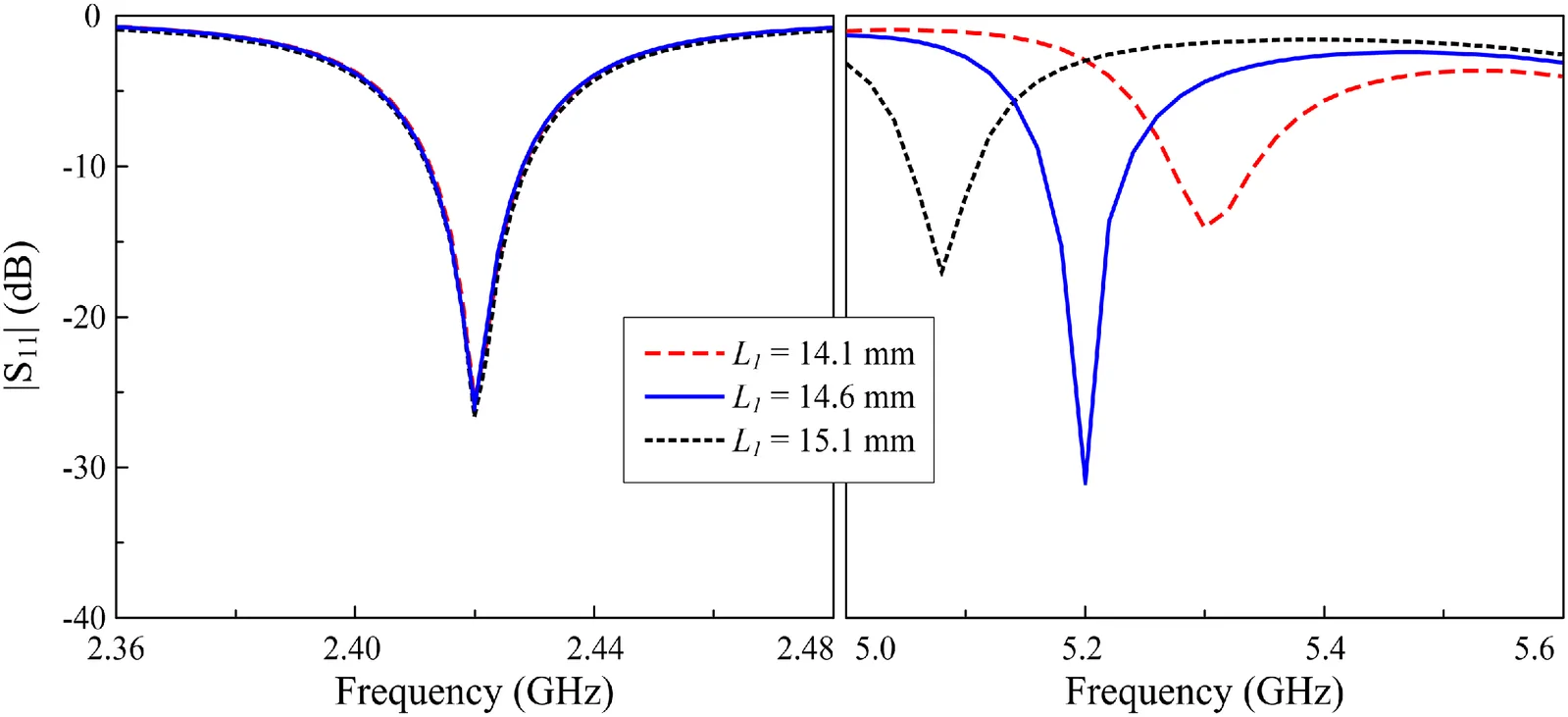
Simulated reflection coefficient for variations in *L*_1_.

**Fig 9:**
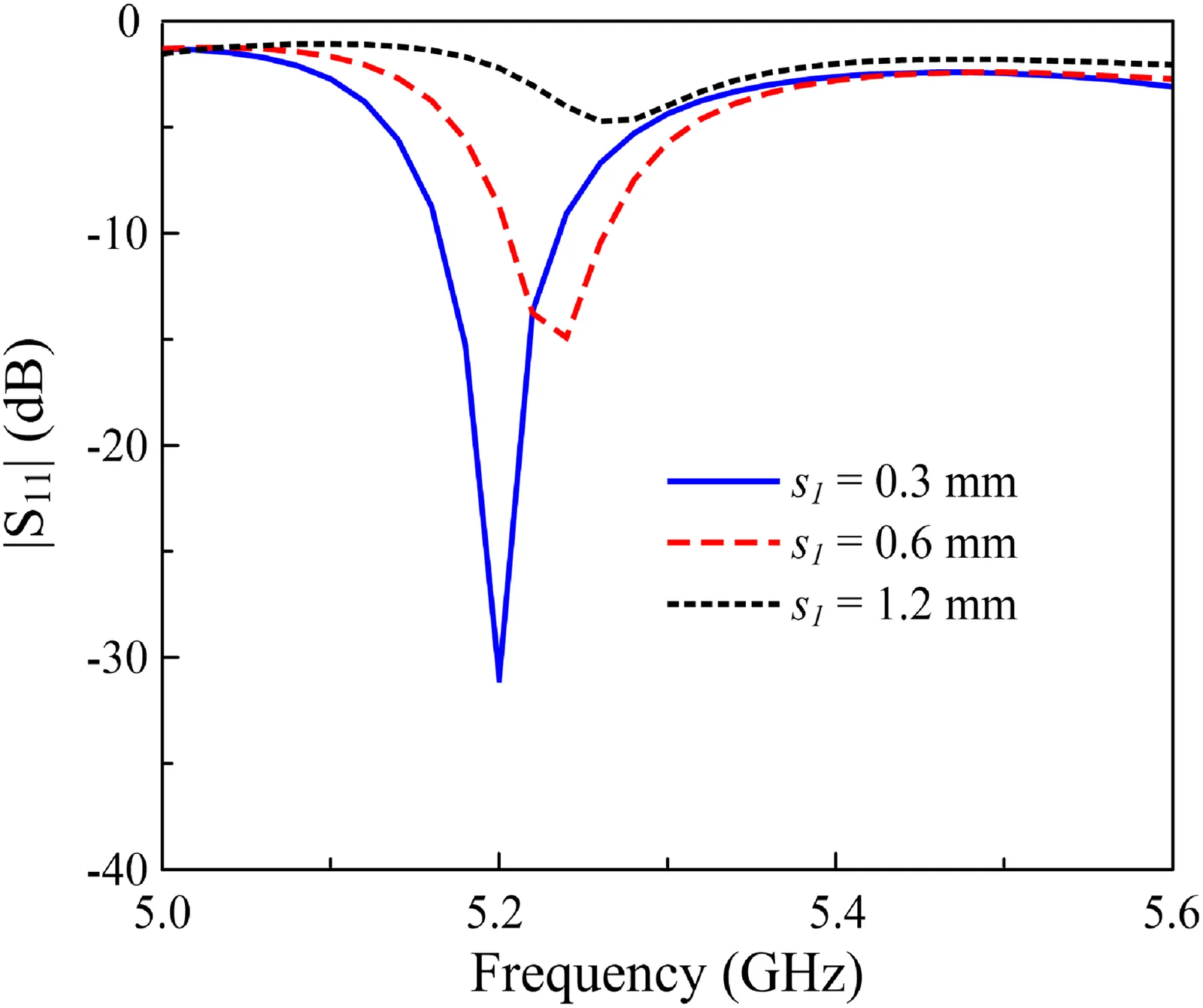
Simulated reflection coefficient for variations in *s*_1_.

## Proposed tri-band antenna

### Antenna configuration

The proposed tri-band antenna is obtained by placing a second parasitic patch on the opposite side of the driven radiator, as shown in Fig 10. The central patch retains 2.4-GHz resonance. The longer and shorter parasitic patches generate the 5.2- and 6.0-GHz resonances, respectively. Their symmetric placement around the fed patch preserves a compact aperture while providing separate upper-band current paths. The optimized dimensions are *L* = 13, $l_{i}=9.4$, $w_{i}=1.8$, *g* = 0.22, *d* = 3.8, $l_{s}=6.0$, $w_{s}=1.0$, *a* = 2.7, *b* = 0.5, $d_{v}=0.4$, $s_{1}=s_{2}=0.4$, *L*_1_ = 14.6, *W*_1_ = 8, *L*_2_ = 12.6, and *W*_2_ = 8 mm.

**Fig 10:**
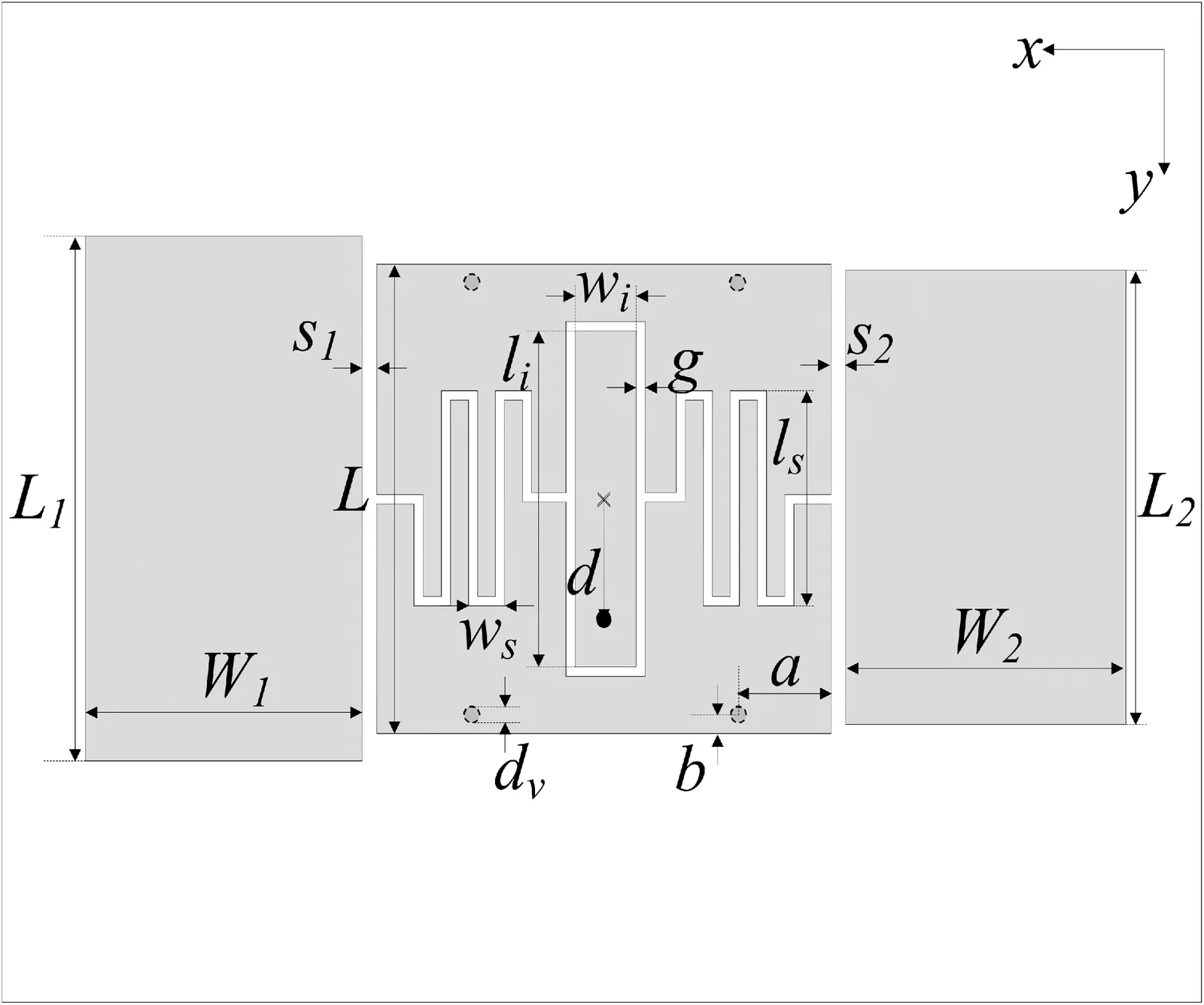
Geometry of the proposed compact tri-band antenna.

### Tri-band performance

The simulated reflection coefficient and peak realized gain are shown in Fig 11. Three resonances with |*S*_11_| below −10 dB are obtained over 2.41–2.43, 5.18–5.29, and 5.90–6.00 GHz. The corresponding peak realized gains are 3.3, 6.0, and 7.3 dBi. The effect of the highest-band patch is examined in Fig 12 by varying *L*_2_. Increasing *L*_2_ shifts only the 6.0-GHz resonance appreciably, whereas the 2.4- and 5.2-GHz responses remain nearly unchanged. This selective sensitivity demonstrates the weak interaction among the three resonant paths and enables sequential band-by-band optimization.

**Fig 11:**
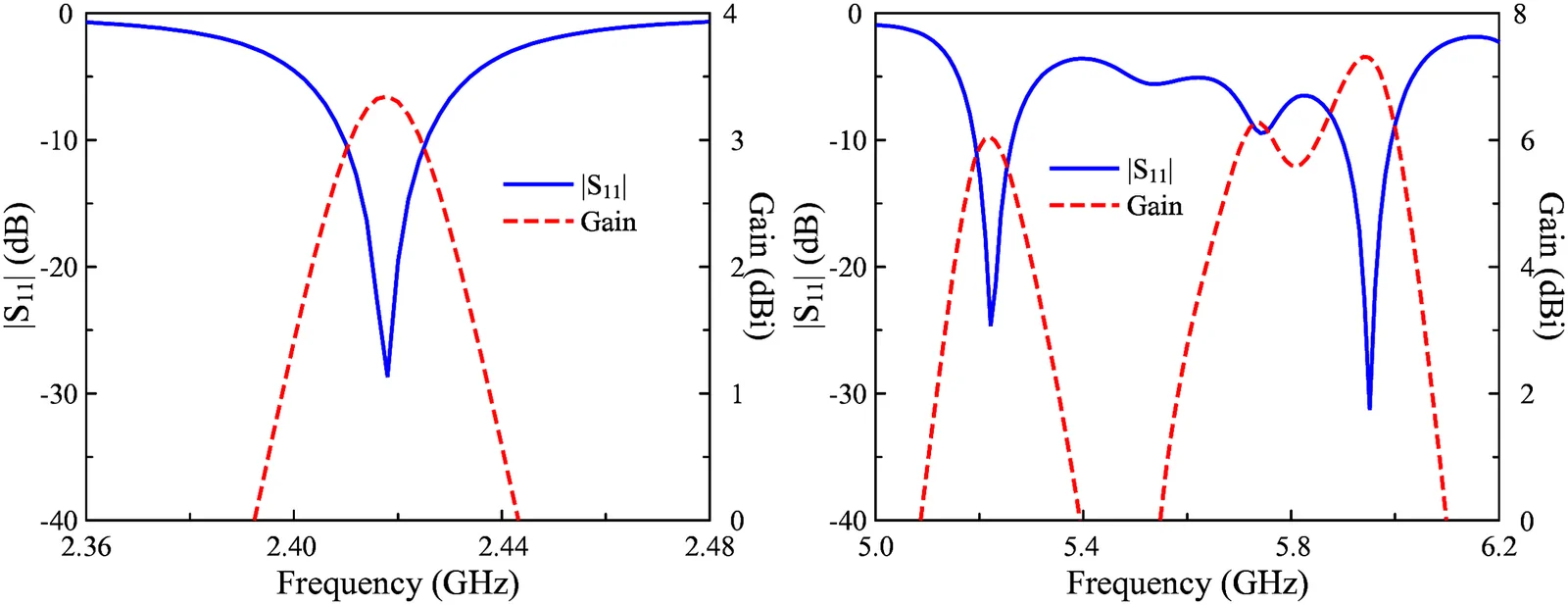
Simulated reflection coefficient and peak realized gain of the tri-band antenna: for the low band (left) and high band (right).

**Fig 12:**
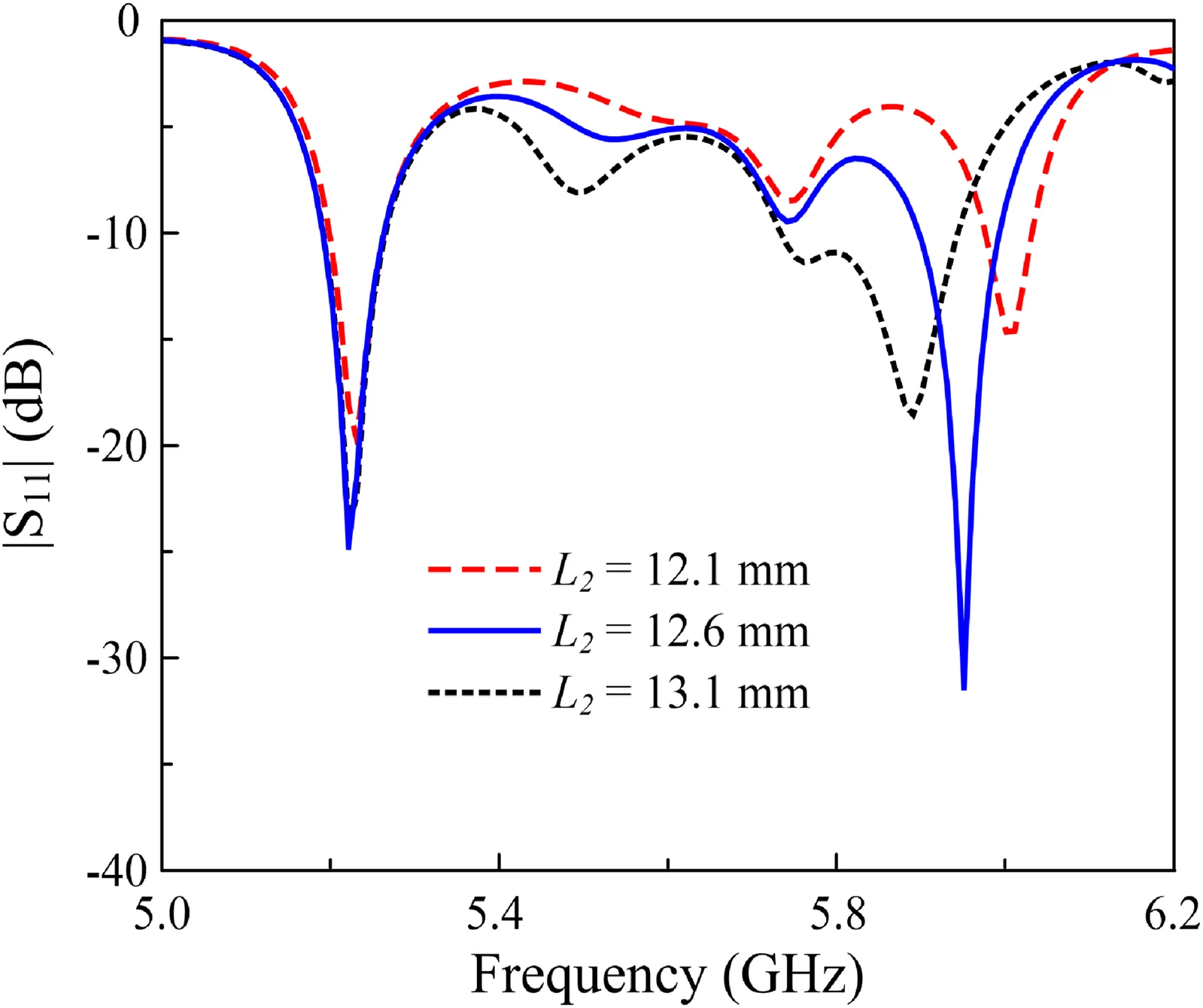
Simulated reflection coefficient of the tri-band antenna for variations in *L*_2_.

The simulated surface-current distributions in Fig 13 further clarify the operating mechanism. At 2.4 GHz, the current is concentrated primarily on the central loaded patch. At 5.2 GHz, the dominant current flows on the longer parasitic patch, whereas at 6.0 GHz it is concentrated on the shorter parasitic patch. The localized current distributions corroborate the parametric analysis and confirm that each operating band is governed by a distinct radiator.

**Fig 13:**
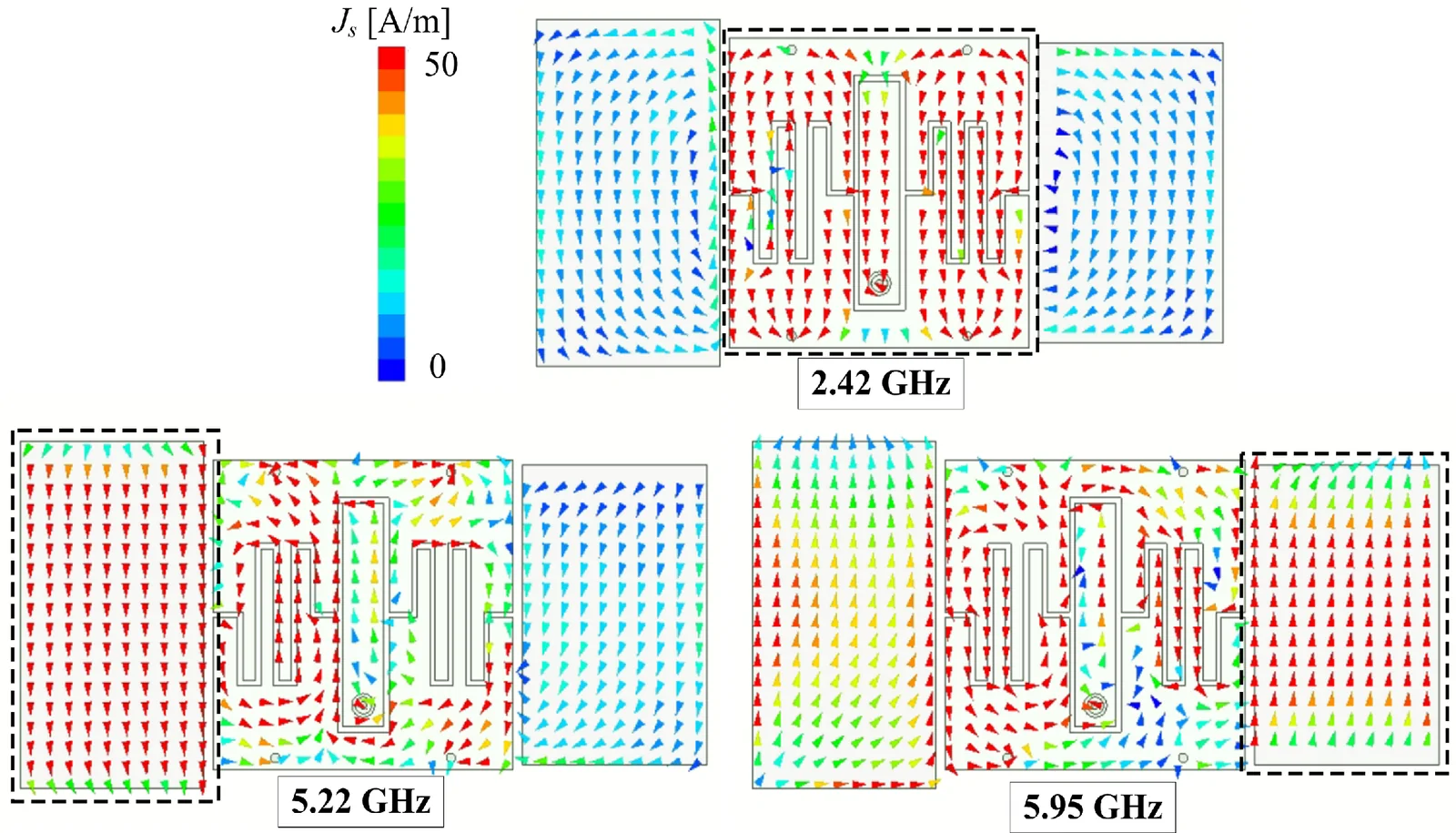
Simulated surface-current distributions at 2.4, 5.2, and 6.0 GHz.

### Design procedure

Based on the preceding analysis, the design procedure can be summarized as follows:

**Step 1: Design the lowest-band radiator.** Select a square patch with an initial side length of approximately 0.2 effective wavelength at the target frequency. Introduce narrow capacitive gaps, shorting vias, and meandered sections to reduce the resonant length. Adjust the gap width *g* and meander length $l_{s}$ to place the lowest resonance at the required frequency, as indicated by Fig 3.
**Step 2: Introduce the middle-band radiator.** Place a rectangular parasitic patch adjacent to the driven patch and choose its initial length near one-half of the effective wavelength at the target middle frequency. Tune the patch length *L*_1_ to set the resonance and the coupling gap *s*_1_ to optimize the impedance matching, as shown in Figs 8 and 9.
**Step 3: Introduce the highest-band radiator.** Add a second rectangular parasitic patch on the opposite side of the driven patch. Select its initial length near one-half of the effective wavelength at the highest target frequency and then adjust *L*_2_ and *s*_2_ to control the highest-band resonance and matching. Finally, re-optimize the three radiators jointly to compensate for the residual inter-element coupling.

## Experimental validation

A prototype was fabricated and measured to validate the proposed concept, as shown in Fig 14. The SMA connector is located beneath the fully metallized ground plane and is therefore physically separated from the radiating patches. Consequently, its direct influence on the radiation characteristics is expected to be limited. Overall, the measured tri-band response and gain trends are consistent with the simulations. The differences are attributed primarily to fabrication tolerances and alignment uncertainty in the far-field measurement setup.

**Fig 14:**
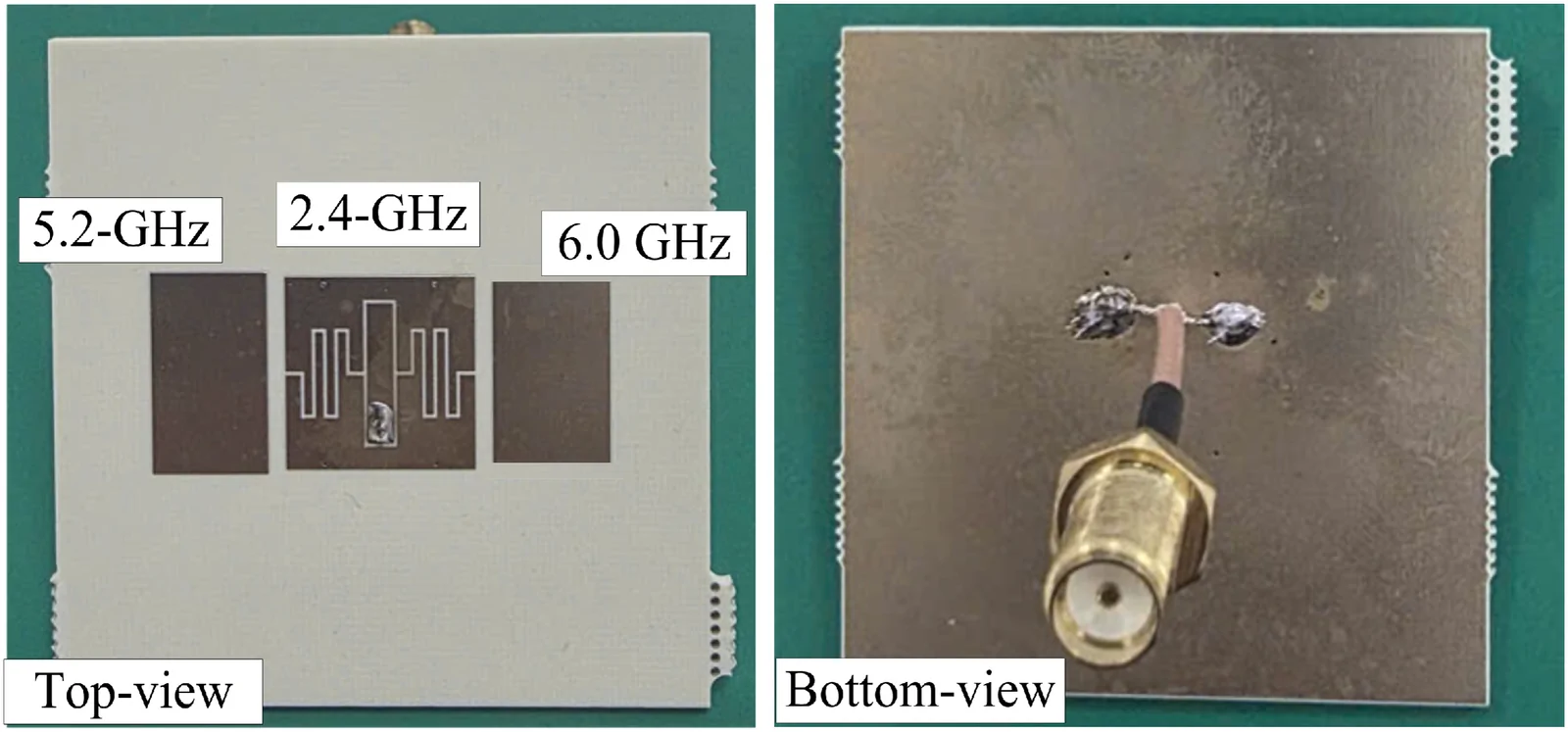
Fabricated prototype: top and bottom views.

Fig 15 compares the simulated and measured reflection coefficients and broadside realized gains. The measured −10-dB bands are 2.42–2.44, 5.24–5.33, and 5.96–6.06 GHz. These correspond to absolute bandwidths of 20, 90, and 100 MHz, and fractional bandwidths of approximately 0.82%, 1.70%, and 1.66%, respectively. Although the antenna is not intended to cover the complete 2.4-, 5-, and 6-GHz WLAN allocations, each band supports channel-specific Wi-Fi 6/6E operation. The lower band accommodates a selected 20-MHz channel near 2.43 GHz. The middle and upper bands can each support an 80-MHz channel, or multiple 20- and 40-MHz channels, near 5.2 and 6.0 GHz, respectively. The measured broadside realized gain varies from 2.5 to 3.1, 4.8 to 5.8, and 5.8 to 6.8 dBi. Here, a strong miniaturization at 2.4 GHz reduces the effective radiating aperture, while the electrically small ground plane increases back radiation, resulting in a lower gain. At the higher bands, the parasitic patches operate near their fundamental half-wavelength modes and therefore provide higher gain. Further simulation data also indicates that at the lower band, the efficiency is approximately 60%, which is acceptable considering the strong 61% miniaturization of the 2.4-GHz radiator. Meanwhile, the upper-bands achieve efficiencies of approximately 80%. As shown in Fig 16, the measured patterns retain broadside directional behavior in both principal planes at all investigated frequencies. It is noted that the cross-polarization level is lower at the lower band and increases at the higher bands, mainly due to differences in current symmetry and the active radiating elements at each frequency. In addition, the asymmetric excitation of the upper-band parasitic patches causes a slight beam tilt away from the broadside direction.

**Fig 15:**
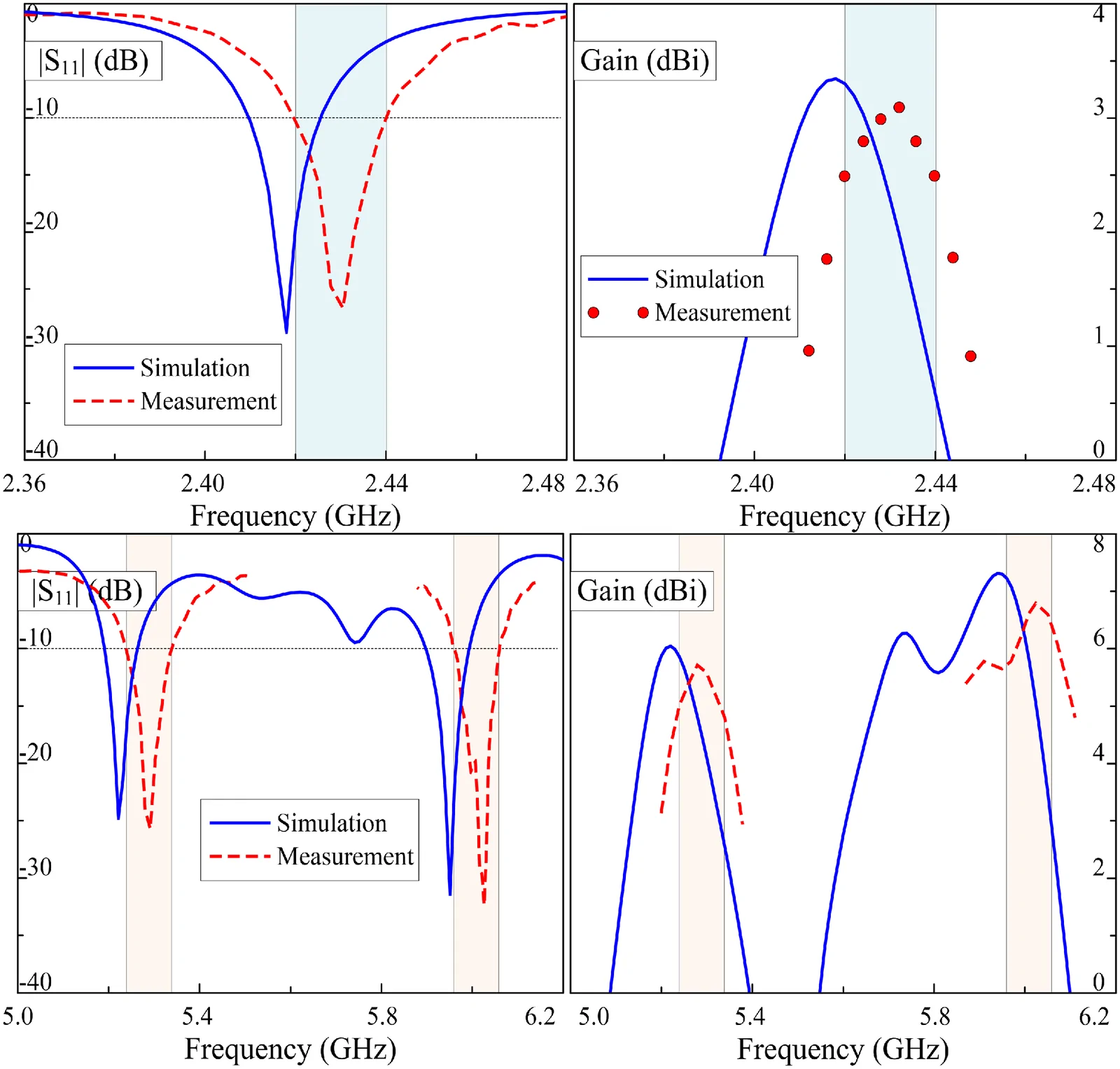
Simulated and measured reflection coefficients and broadside realized gains across the low band and upper bands, respectively shown in the upper and lower panels.

**Fig 16:**
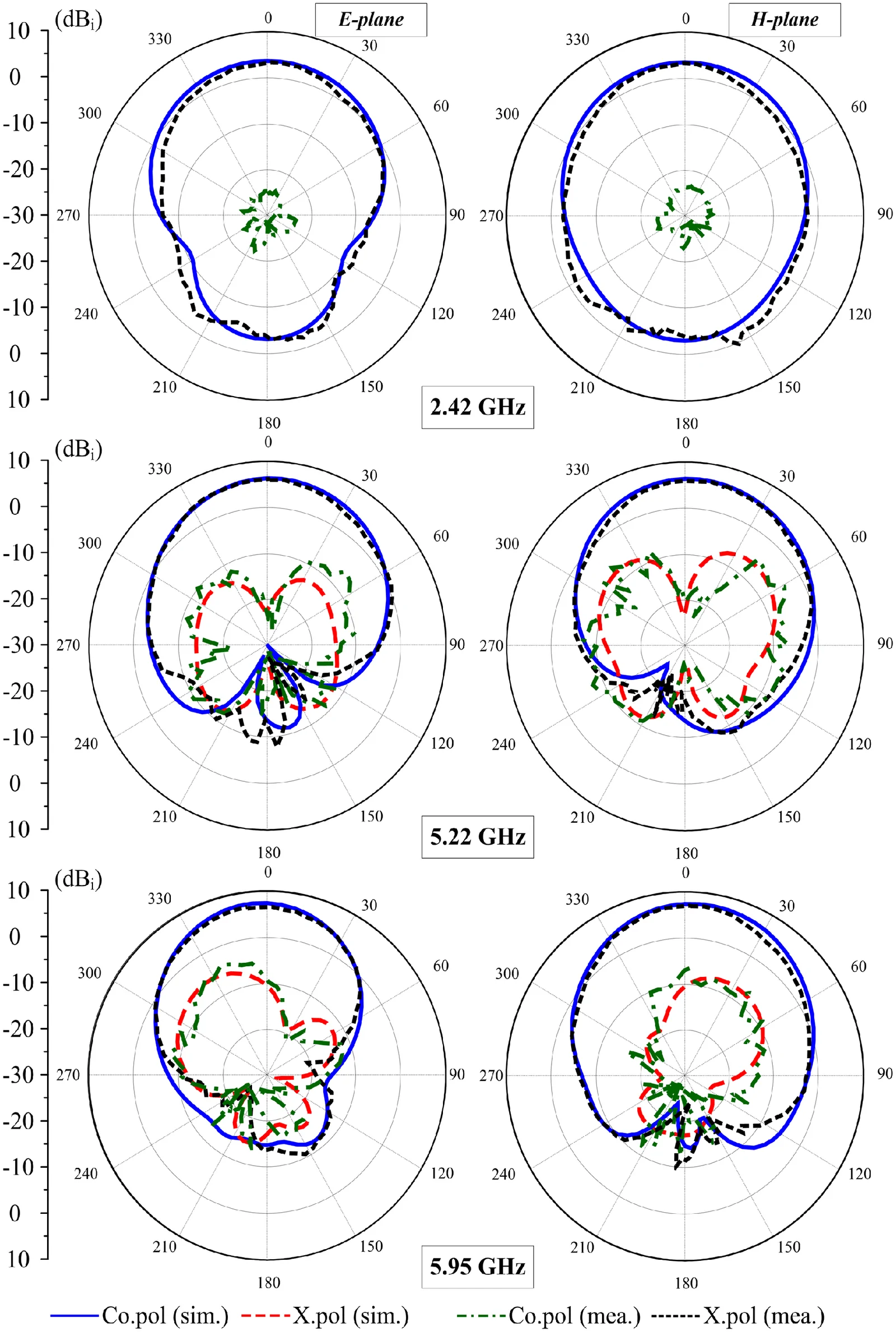
Simulated and measured radiation patterns at 2.4, 5.2, and 6.0 GHz.

## Performance comparison

Table 1 compares the proposed antenna with representative tri-band directional designs. The proposed antenna operates at 2.4, 5.2, and 6.0 GHz with a frequency ratio $f_{h}/f_{l}=2.50$, thereby providing direct channel-specific coverage of the Wi-Fi 6/6E bands. References [19–21] achieve substantially larger ratios of 4.67–25 by combining microwave and millimeter-wave resonances. These shared-aperture designs provide widely separated services and high millimeter-wave gain, but require five or six layers, larger normalized footprints, and higher profiles, which increase fabrication complexity and integration cost.

**Table 1:** Comparison with reported tri-band directional antennas.

Ref.	Profile (λ)	Footprint (λ)	Layers	Bands (GHz)	$f_{h}/f_{l}$	Peak gain (dBi)	Primary application and Wi-Fi 6/6E relevance
*Microwave/mmWave shared-aperture designs with very large frequency ratios*							
[19]	0.10	1.12 × 1.12	6	2.4 / 5.0 / 60	25.0	9.8 / 7.9 / 8.4	Wi-Fi 6 at 2.4/5 GHz + 60-GHz WiGig; no 6-GHz Wi-Fi 6E
[20]	0.07	1.21 × 0.87	6	5.9 / 28 / 39	6.61	8.0 / 11 / 11.3	V2X + 5G mmWave; not intended for Wi-Fi 6/6E
[21]	0.04	0.65 × 0.56	5	6.0 / 8.0 / 28	4.67	6.8 / 6.1 / 6.0	Sub-6/mmWave 5G/6G; only the 6-GHz band overlaps Wi-Fi 6E
*All-sub-6-GHz tri-band designs*							
[22]	0.05	0.78 × 0.78	3	2.6 / 3.6 / 5.3	2.04	7.2 / 6.0 / 4.4	WLAN/WiMAX; only the highest band is within Wi-Fi 6
[23]	0.04	0.48 × 0.48	3	1.2 / 1.6 / 2.3	1.92	5.0 / 5.0 / 7.0	GNSS L5/L1 + SDARS; no Wi-Fi 6/6E coverage
[24]	0.03	0.55 × 0.34	2	2.4 / 3.5 / 5.8	2.42	6.2 / 5.5 / 8.4	Wi-Fi 6 at 2.4/5.8 GHz + 3.5-GHz 5G; no 6-GHz Wi-Fi 6E
[26]	0.01	0.33 × 0.27	1	2.0 / 3.5 / 5.2	2.60	1.7 / 1.8 / 5.4	UMTS/WiMAX/5.2-GHz ISM; not tri-band Wi-Fi 6/6E
[27]	0.02	0.44 × 0.40	1	3.6 / 4.8 / 5.8	1.61	5.0 / 6.9 / 5.8	Chinese 5G bands; only upper band overlaps 5-GHz Wi-Fi
Prop.	0.012	0.36 × 0.36	1	2.4 / 5.2 / 6.0	2.50	3.1 / 5.8 / 6.8	Channel-specific operation in the 2.4-, 5-, and 6-GHz Wi-Fi 6/6E

The all-sub-6-GHz antennas in [22–24,26,27] are structurally simpler, but they either exhibit a smaller frequency ratio or do not simultaneously address the 2.4-, 5-, and 6-GHz Wi-Fi bands. In contrast, the proposed antenna combines a 0.36λ × 0.36λ footprint, a 0.012λ profile, single-layer construction, one-port excitation, and measured gains of 3.1, 5.8, and 6.8 dBi. Its principal contribution is therefore the balanced combination of Wi-Fi 6/6E-oriented frequency coverage, a relatively large all-sub-6-GHz frequency ratio, compact dimensions, directional radiation, and nearly independent resonance control.

## Conclusion

A compact single-layer, single-feed tri-band directional antenna has been developed for channel-specific Wi-Fi 6/6E operation. Combined capacitive and inductive loading reduces the resonant length of the 2.4-GHz driven patch by approximately 61%, while two adjacent parasitic patches generate independently controllable resonances at 5.2 and 6.0 GHz. The fabricated 0.36λ × 0.36λ × 0.012λ prototype achieves measured impedance bandwidths of 20, 90, and 100 MHz and peak broadside realized gains of 3.1, 5.8, and 6.8 dBi, respectively. The measured results confirm broadside directional radiation in all three bands and demonstrate a favorable tradeoff among frequency ratio, compactness, gain, and structural simplicity. The narrow lower-band bandwidth is a direct tradeoff associated with the strong size miniaturization. Future work will focus on bandwidth enhancement and possible practical approaches include: (i) introducing a closely coupled additional low-band parasitic resonator; and (ii) reducing the miniaturization ratio or employing a thicker, lower-permittivity substrate to decrease the antenna quality factor, at the expense of increased size or profile.
